# Nano Pt/TiO_2_ photocatalyst for ultrafast production of sulfamic acid derivatives using 4-nitroacetanilides as nitrogen precursor in continuous flow reactors

**DOI:** 10.1007/s11356-023-25968-9

**Published:** 2023-02-21

**Authors:** Yasser M. A. Mohamed, Yasser A. Attia

**Affiliations:** 1 Photochemistry Department, National Research Center, Dokki, Giza, 12622 Egypt; 2 National Institute of Laser Enhanced Sciences, Cairo University, Giza, 12613 Egypt

**Keywords:** Sulfur oxides, Photocatalytic conversion, Pt/TiO_2_, Nanoparticles, Organo-sulfonic acid derivatives

## Abstract

The design of reactors based on high performance photocatalysts is an important research in catalytic hydrogenation. In this work, modification of titanium dioxide nanoparticles (TiO_2_ NPs) was achieved by preparation of Pt/TiO_2_ nanocomposites (NCs) through photo-deposition method. Both nanocatalysts were used for the photocatalytic removal of SOx from the flue gas at room temperature in the presence of hydrogen peroxide, water, and nitroacetanilide derivatives under visible light irradiation. In this approach, chemical deSOx was achieved along with protection of the nanocatalyst from sulfur poising through the interaction of the released SOx from SOx-Pt/TiO_2_ surface with p-nitroacetanilide derivatives to produce simultaneous aromatic sulfonic acids. Pt/TiO_2_ NCs have a bandgap of 2.64 eV in visible light range, which is lower than the bandgap of TiO_2_ NPs, whereas TiO_2_ NPs have a mean size of 4 nm and a high specific surface area of 226 m^2^/g. Pt/TiO_2_ NCs showed high photocatalytic sulfonation of some phenolic compounds using SO_2_ as a sulfonating agent along with the existence of p-nitroactanilide derivatives. The conversion of p-nitroacetanilide followed the combination processes of adsorption and catalytic oxidation–reduction reactions. Construction of an online continuous flow reactor–high-resolution time-of-flight mass spectrometry system had been investigated, realizing real-time and automatic monitoring of completion the reaction. 4-nitroacetanilide derivatives (**1a-1e)** was converted to its corresponding sulfamic acid derivatives (**2a–2e)** in 93–99% isolated yields of within 60 s. It is expected to offer a great opportunity for ultrafast detection of pharmacophores.

**Supplementary Information:**

The online version contains supplementary material available at 10.1007/s11356-023-25968-9.

## Introduction

Air pollution has been identified as a major environmental health risk factor, causing an estimated 3.7 million early deaths globally (Abdelsalam et al. 2020; Khallaf 2011). Emissions of the air pollutants are mainly caused by agricultural activities and combustion of fuels in industrialized societies and are together and separately causing problems with human health, acidification, eutrophication, vegetation damages, and corrosion (Spix et al. 1998). Even though air pollution problems currently are more severe on other continents they still cause problems (Ayturan and Dursun 2018). Contaminated air, as is well known, comprises a wide range of chemical components, including dust, toxic gases, greenhouse gases, and metal salts. Sulfur dioxide (SO_2_) as one of polluted gas, the most frequent form of sulfur oxides (SOx) in the lower atmosphere, is a colorless gas with a pungent and disagreeable odor that can be detected by taste and smell in concentrations ranging from 1000 to 10,000 g/m^3^ (Krishnan et al. 2013; Nanayakkara et al. 2014; Flores et al. 2017). Sulfur trioxide (SO_3_), a sulfur oxide that is immediately discharged into the atmosphere or generated from sulfur dioxide, is swiftly converted to sulfuric acid (Ackermann et al. 1998; Cheremisinoff 2002). Sulfur oxide pollution has a negative impact on the ecosystem, especially forests and agricultural crops. Plants in close proximity to pollution sources are particularly sensitive. Even after a short exposure to high levels of sulfur dioxide, foliar necrosis can occur. The effects of forest ecosystems vary greatly depending on soil type, plant species, meteorological conditions, insect numbers, and other unknown factors (Pan 2019). As for critical levels of gaseous sulfur dioxide, it is not of practical importance that sulfur inputs in mist and rain may also contribute to indirect effects on vegetation (i.e., soil-mediated) and thus may also be included in critical load calculations. By reducing the pH of the water, acid depositions can affect freshwater lake and stream ecosystems. Lakes with low buffering capacity, which could assist in acid rain neutralization, are particularly vulnerable. Few fish species can withstand substantial pH fluctuations, and lakes that are affected may become completely barren of fish life. Acidification reduces the diversity and quantity of other animal and plant species (Chen et al. 2007). By scattering light, sulfate aerosols, which are formed from sulfur dioxide in the atmosphere, can affect visibility (Cheremisinoff 2001). The corrosion of iron, steel, and zinc is accelerated by sulfurous acid, which is generated when sulfur dioxide reacts with moisture. Sulfur oxides react with copper to form a green patina of copper sulfate on the metal’s surface (Clarelli et al. 2014).

The most possible solutions for of SOx emissions (Godish 1991; Carey et al. 2006; Mills et al. 2007) are the following: (i) the use of low sulfur fuel oil; (ii) exhaust gas scrubber technology. In this technology, a scrubbing liquid is prepared from fresh water and sodium hydroxide which reduces the SOx to 95%. Then, the effluent water tends to treatment processes before it can be discharged overboard; (iii) Cylinder lubrication for engines along with efficient control systems to neutralize the sulfur in the fuel and reduce SOx emissions from the engine. It was worthy to note that each solution is expensive. Hence, an innovative approach is required (Moors et al. 2017; Wang et al. 2017; Rabiee and Mahanpoor 2018). Today, the catalysts possessing large surface area have been progressed to become one of the most important branches of advanced materials, organic chemistry, and nanotechnology. These catalysts have certain unique characteristics, such as stability in air and water, ease of handling, and even reusability (Shang et al. 2002; Wang and You 2018; Su et al. 2013). In addition, sulfonation of aromatic compounds is a very important chemical reaction. Along with nitration, halogenation, acylation, and alkylation, it belongs to the well-known class of electrophilic aromatic substitution (SEAr) reactions. In large industrial applications such as medicines, detergents, surfactants, dyes, and pesticides, sulfonation is a significant chemical step. Sulfur trioxide (SO_3_), oleum, sulfuric acid, and chlorosulfuric acid are the most regularly utilized sulfonating agents (Smith and Tatchell 1969; Roberts 1998; Chen et al. 2012). Acute SO_2_ exposure (single exposures) causes rapid bronchial constriction, airway narrowing, increased pulmonary resistance, and enhanced airway responsiveness (Baltrusaitis et al. 2011). As a result, eliminating SOx is very important, especially if it can be used in eco-friendly synthesis of organo-sulfonic acid derivatives. Various methods may convert SO_2_ to other compounds and/or remove it from the atmosphere once it is released into the atmosphere (Wang et al. 2016; Amini et al. 2019). The removal of SO_2_ from the atmosphere is aided by processes such as oxidation, wet deposition, dry deposition, absorption by vegetation and soil, solubility into water, and others. Today, the development of green techniques in chemical transformations (Attia and Mohamed 2022; Attia and Abdel-Hafez 2022) using recyclable nanocatalyst to afford high selectivity and catalytic performance has attracted great attentions (Taha et al. 2022; Attia and Abdel-Hafez 2021; Al-Musawi et al. 2022a; Attia 2016). Moreover, the regeneration/recovery of the initial photocatalytic activity through combining photocatalysis and nonthermal plasma was explored in several studies (Hassani et al. 2017; Abou Saoud et al. 2017). Titanium dioxide is among the most prevalent photo-nanocatalysts (TiO_2_) (Li et al. 2011; Liu et al. 2014; Bellardita et al. 2017; Eltohamy et al. 2022; Al-Musawi et al. 2023) due to its wide bandgap (3.2 eV), long-term photostability, low cost, low operational temperature, strong oxidation capability, non-toxic nature, and strong resistance to chemical and photocorrosion (Tada-Oikawa et al. 2016; Tsuneyasu et al. 2016). However, TiO_2_ NPs have some drawbacks, including a maximum wavelength in the ultraviolet, a tiny surface area, and rapid recombination of photogenerated electrons and holes (Attia and Altalhi 2017). By shifting its light absorption to the visible region and narrowing its bandgap energy, doping Titania with metal and nonmetal elements is one of the most active ways to improve its efficiency and overcome the shortcomings. Recently, noble metal doping TiO_2_ NPs were used for the NO_x_ conversion into nitric acid (HNO_3_) under visible light irradiation (Abdelsalam et al. 2020) and TiO_2_ nanoparticles were used for dye photodegradation and H_2_ production (Attia and Altalhi 2017). Currently, the combination between nanocatalysis field and continuous flow technology has attracted the attentions of scientists (Liu et al. 2012; El Kadib et al. 2009; Al-Musawi et al. 2022b). Therefore, efforts shall be made with the design of novel reactors to push forward the application of photocatalysts based on TiO_2_ nanoparticles in organic transformations (Mohamed et al. 2021). Today, the Z-scheme photocatalysts showed high performance for contaminants elimination due to their visible light response and their strong redox ability (Hassani et al. 2021). Herein, we report the first trial to design a continuous flow reactor using Pt/TiO_2_ NCs for ultrafast conversion of 4-nitroacetanilide derivatives to sulfamic acids through catalytic reduction reaction. These methods open the access to use the Pt/TiO_2_ nanocatalyst as dual function material in which the use of SOx gas in design high-valued pharmaceutical products in spontaneous reaction process. In addition, the synthesized products had been characterized using different analytical tools. The main objective of this research proposal is to use new advanced techniques for the production of pharmaceutical products included: (i) Design new synthetic routes by using safe reagents and solvents; (ii) Improve the yield of the products and selectivity of the reactions in place of traditional industrial process.

## Materials and methods

TiCl_4_, PtCl_2_, and 4-nitroaniline derivatives and ethanol were purchased from Sigma-Aldrich Company and were used without further purification. The N-acylation of 4-nitroaniline is a fundamental organic reaction that was used for the production of p-nitroacetanilide derivatives (Greene and Wuts 2007). Pt/TiO_2_ NCs and TiO_2_ NPs as photocatalysts were also according to literature procedures, respectively (Attia and Altalhi 2017; Bellardita et al. 2017). These synthesized nanoparticles were characterized by XRD, TEM, DRS, FTIR, BET, and UV–VIS spectrometer. Then the prepared photocatalysts were used in ecofriendly sulfonation of organic materials to synthesize organo-sulfonic acid derivatives as fine and pharmaceutical products. ^1^H and ^13^C NMR spectra for the sulfamic derivatives were recorded on Bruker Avance DPX (300 MHz for ^1^H and 75 MHz for and ^13^C, respectively). The chemical shift (δ) data were determined in parts per million (ppm) downfield from tetramethylsilane using DMSO-*d*_*6*_ as a partially deuterated NMR solvent. High-resolution mass spectrometry (HRMS) analysis was measured by 6230 Series Accurate-Mass Time-Of-Flight (TOF) liquid chromatography (LC)/MS system.

### Preparation of TiO_2_ NPs

TiCl_4_ was mixed with deionized water (1:10) under vacuum condition with stirring for 30 min at 80 °C. The solution was left overnight to precipitate, and then the prepared precipitate was filtrated. TiO_2_ NPs were obtained after calcination for 4 h at 550 °C (Attia and Altalhi 2017).

### Preparation of Pt/TiO_2_ NCs

To a suspension solution of TiO_2_ NPs (0.5 g) in 100 mL of distilled water and ethanol (50 mL), appropriate PtCl_2_ (corresponding to 0.5 wt% metal loading) were added. Then, the mixture was placed in a 1.5-L cylindrical photoreactor equipped with a medium pressure Hg lamp (500 W, 400–790 nm. 47–63 HZ). The suspensions were irradiated for 6 h (Bellardita et al. 2017).

### Photocatalytic activity assessment toward SO_2_ removal and synthesis of organo-sulfonic acid derivatives

An amount equal of the selected photo-nanocatalysts, i.e., Pt/TiO_2_ NCs or TiO_2_ NPs (5 mg or 7.5 mg) was loaded in a mixture of (25 mL deionized water and 25 mL ethanol) and 0.15 g of the organic material (p-nitroacetanilide derivatives) within a ratio (1/3 or 1/2, w/w), and then 10 ppm concentration of SOx gas was passed through the reaction solution with 1.0 L/min flow rate for 10 min at room temperature. Then, the output data of SO_2_ outflow concentration was recorded. The setup included a SOx gas supply, a reactor, and analytical system. The reaction was carried out at room temperature (25 °C) under visible light irradiation. SO_2_ was analyzed with a gas analyzer (Thermo Scientific SO_2_ analyzer 43i).

### Experimental setup 

**Fig. 1:**
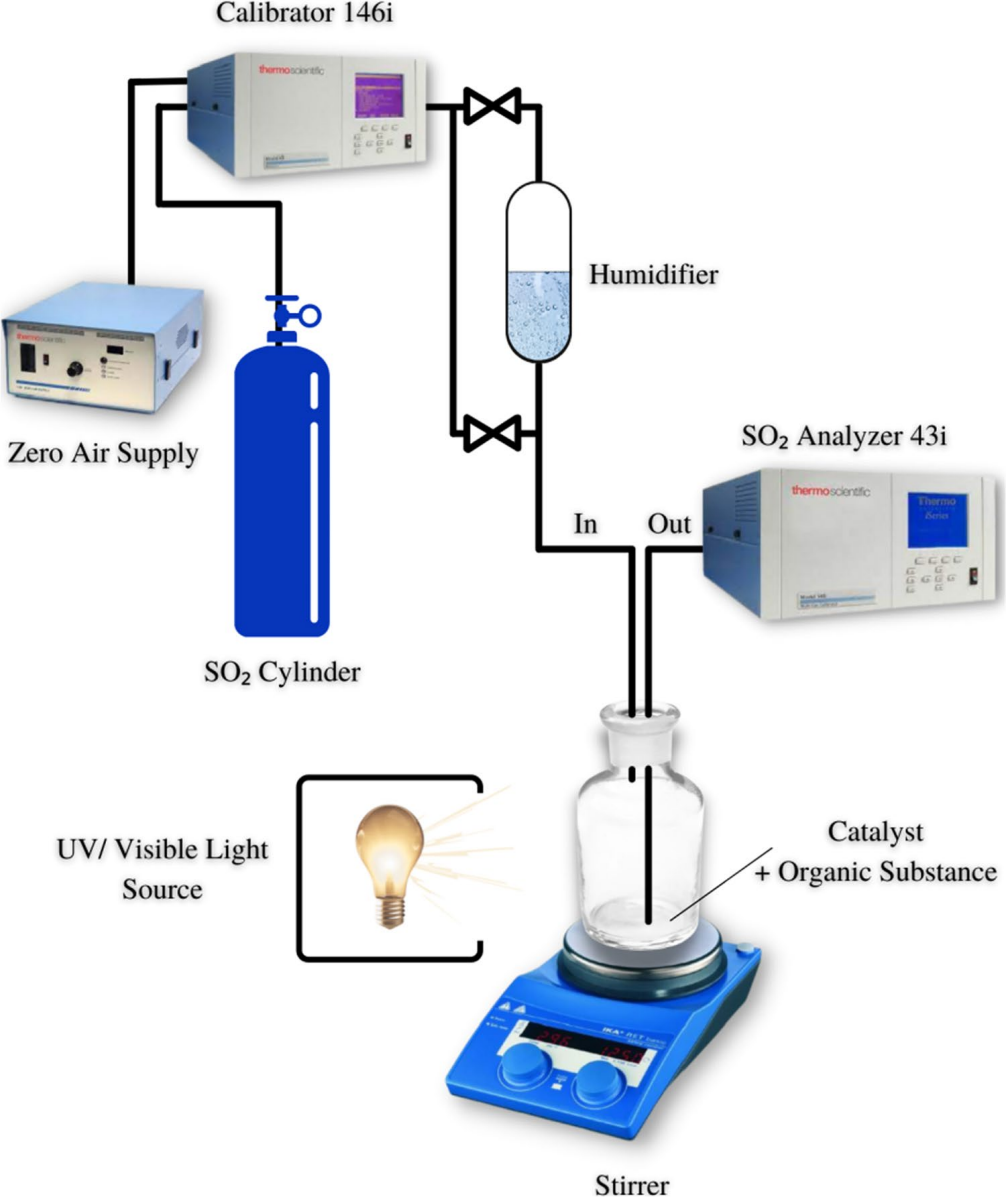
Experimental setup of SOx conversion

The scope of the test setup is to determine the air-purification performance of the photocatalyst, titanium dioxide, or titanium dioxide nanocluster, by continuous exposure of a specimen to the model air pollutant under illumination with visible light. A Thermo Scientific Company Model 1160 zero air generator was used to supply air. A SO_2_ primary cylinder (100 ppm) and zero air stream were mixed to a specific concentration by a Thermo Environmental Instruments Inc. Model 146 i dynamic gas calibration system. The gas flow and inlet concentration were calibrated by a gas blender of the calibrator. Flow rate of the gas stream was 1.0 L min^−1^ and SO_2_ concentration 10 ppm. The prepared catalyst was loaded into a 500-mL cylindrical glass filled with 25 mL of de-ionized water, 25 mL of ethanol, and 0.5 mL of H_2_O_2_ with stirring in addition to 0.15 g of the starting organic material (Fig. 1). The reaction mixture was irradiated with Vis-lamp (20 W, SANKYO DENKI, Japan) located outside the reactor. SO_2_ were analyzed with a SO_2_ analyzer (Thermo Scientific, Model 43i-SO_2_ analyzer).

In addition, for online continuous reduction of 4-nitroacetanilide, an online catalytic reactor–high-resolution time-of-flight mass spectrometry system was implemented. Firstly, the direct injection method was used to detect the molecular mass of 4-nitroacetanilide in the reaction solution by HR-TOF–Ms.

### Characterization of dioxadithiine-6-sulfonic acid derivatives

**(4-acetamidophenyl)sulfamic acid (2a)**: ^1^H NMR: δ (ppm) 2.03 (s, 3H, CH_3_), 5.06 (s, br, 1H, SO_3_H), 6.78 (d, 2H, J = 8.4 Hz, 2H), 7.34 (d, 2H, J = 8.4 Hz, 2H), 9.02 (br, s, 1H, NH), 10.63 (br, s, 1H, NH); ^13^C NMR (75 MHz, DMSO-*d*_*6*_): 22.5, 23.8, 26.8, 129.6, 132.4, 135.1, 138.0, 155.9. m/z (HRMS): calcd. for C_8_H_10_N_2_O_4_S: 230.0361, found 231.0741 for [M + H]^+^.

**(4-(2,2,2-trifluoroacetamido)phenyl)sulfamic acid (2b)**: ^1^H NMR: δ (ppm) 5.12 (s, br, 1H, SO_3_H), 6.82 (d, J = 8.4 Hz, 1H), 7.36 (d, J = 8.4 Hz, 1H), 9.08 (s, br, 1H), 10.61 (s, br, 1H); ^13^C NMR (75 MHz, DMSO-*d*_*6*_): 23.8, 116.2 (2xC), 122.1 (2xC), 128.1, 133.5, 138.0, 167.2. m/z (HRMS): calcd. for C_8_H_7_F_3_N_2_O_4_S: 284.0079, found 285.0103 for [M + H]^+^.

**(4-acetamido-3-methoxyphenyl)sulfamic acid (2c):** ^1^H NMR: δ (ppm) 2.11 (s, 3H, CH_3_), 3.64 (s, 3H, OCH3), 5.11 (s, br, 1H, SO_3_H), 6.84–6.88 (m, 1H), 7.31 (s, 1H), 7.42–7.46 (m, 1H), 9.08 (s, br, 1H), 10.32 (s, br, 1H); ^13^C NMR (75 MHz, DMSO-*d*_*6*_): 24.1, 55.2, 101.8, 108.9, 116.3, 122.9, 128.1, 134.6, 152.1, 168.6. m/z (HRMS): calcd. for C_9_H_12_N_2_O_5_S: 260.0467, found 261.1008 for [M + H]^+^.

**(4-acetamido-3-acetylphenyl)sulfamic acid (2d):** ^1^H NMR: δ (ppm) 2.02 (s, 3H, CH_3_), 2.53 (s, 3H, CH_3_), 2.02 (s, 3H), 2.53 (s, 3H), 5.09 (s, br, 1H, SO_3_H), 7.09–7.16 (m, 1H), 7.33 (s, 1H), 7.48–7.51 (m,1H), 9.64 (s, br, 1H), 10.56 (s, br, 1H); ^13^C NMR (75 MHz, DMSO-*d*_*6*_): 24.2, 29.1, 113.1, 121.9, 122.7, 128.3, 133.8, 168.1, 199.7. m/z (HRMS): calcd. for C_10_H_12_N_2_O_5_S: 272.0467, found 273.0692 for [M + H]^+^.

**(4-acetamido-3-fluorophenyl)sulfamic acid (2e):** ^1^H NMR: δ (ppm) 2.15 (s, 3H, CH_3_), 5.02 (s, br, 1H, SO_3_H), 6.72–6.75 (m, 1H), 7.05 (s, 1H), 7.46–7.49 (m, 1H), 9.88 (s, br, 1H), 10.64 (s, br, 1H); ^13^C NMR (75 MHz, DMSO-*d*_*6*_): 24.1, 105.1, 110.5, 111.8, 112.6, 123.9, 134.1, 163.8, 169.3. m/z (HRMS): calcd. for C_8_H_9_FN_2_O_4_S: 248.0267, found 249.1102 for [M + H]^+^.

## Results

### Characterization of the prepared TiO_2_ NPs and Pt/TiO_2_ NCs

**Fig. 2:**
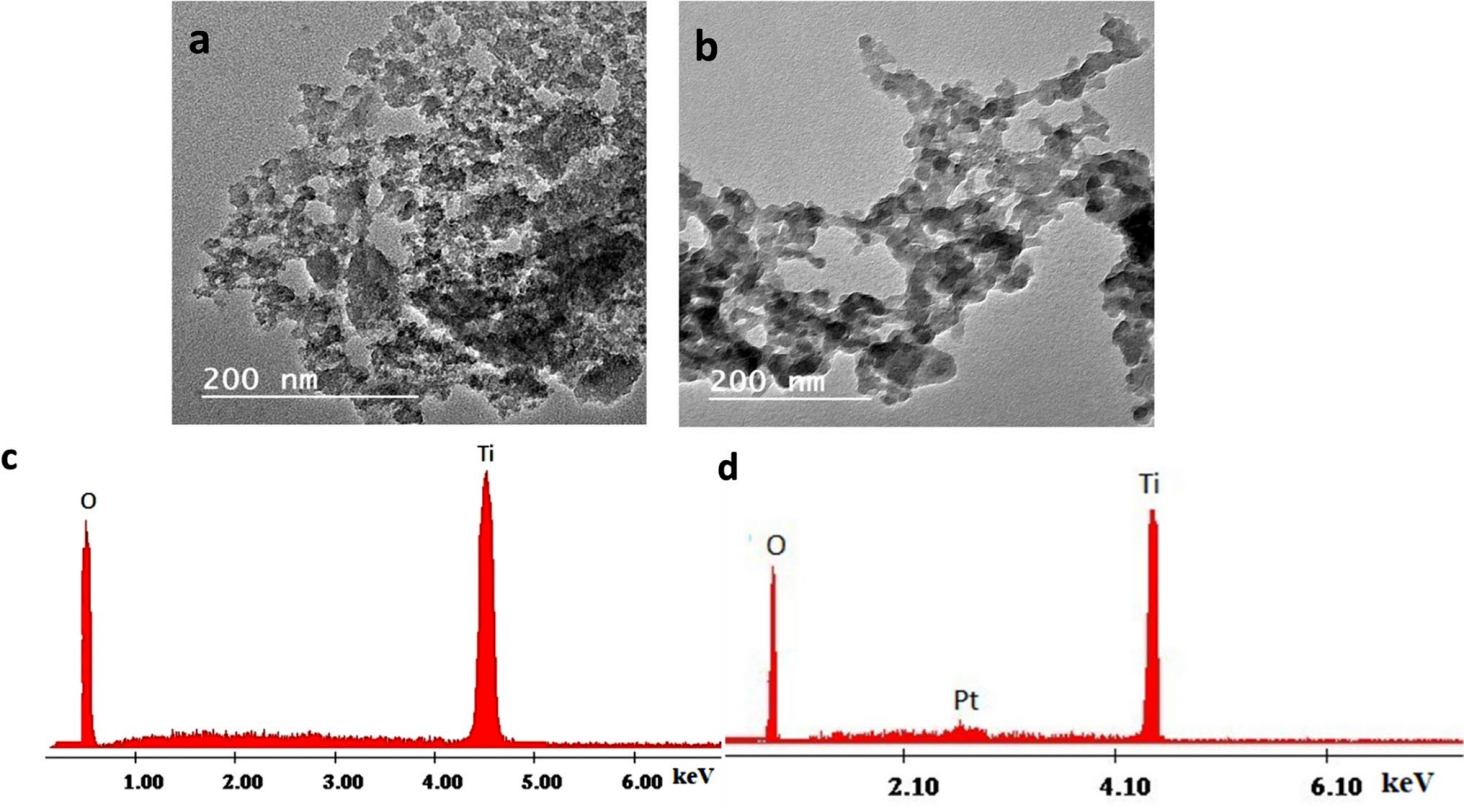
The TEM images of TiO_2_ NPs (**a**) and Pt/TiO_2_ NCs (**b**). EDX of TiO_2_ NPs (**c**) and Pt/TiO_2_ NCs (**d**)

TEM images were used to examine the morphology of the anatase Pt/TiO_2_ NCs and TiO_2_ NPs samples, as shown in Fig. 2. TiO_2_ NPs are found to have a mean size of 4 ± 0.12 nm (Fig. 2a). However, Pt/TiO_2_ NCs with a mean size of 7 ± 1.27 nm were formed (Fig. 1b). An energy dispersive spectrometer (EDS) spectrum was used to examine the chemical compositions of TiO_2_ NPs and Pt/TiO_2_ NCs, which revealed the presence of (Ti and O) with (56.34 wt% and 43.66 wt%) and for Pt/TiO_2_ NCs (Ti, Pt, and O) with (52.61 wt%, 0.57 wt%, and 47.18 wt%), respectively. These findings show that the TiO_2_ in the as-prepared samples is of high purity and homogeneously produced.

**Fig. 3:**
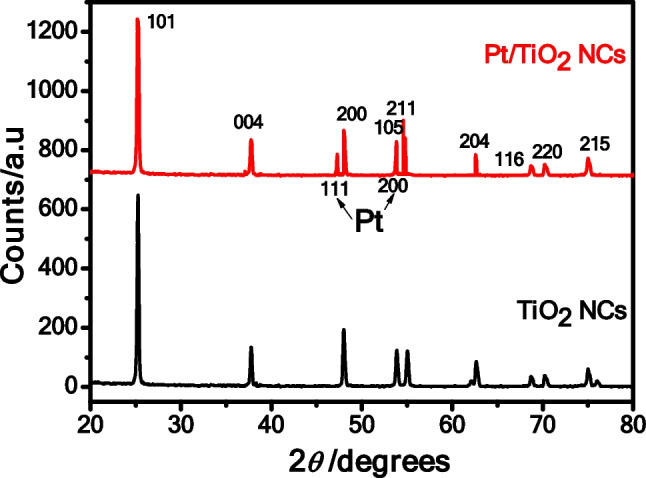
The XRD patterns of TiO_2_ NPs and Pt/TiO_2_ NCs

The crystal structure of TiO_2_ NPs was pure anatase, as revealed by the X-ray diffraction (XRD) patterns in Fig. 3. The optimal peaks for the plane of tetragonal anatase TiO_2_ (JCPDS 21–1272) were found to be 25.50°, 37.92°, 48.00°, 53.71°, 54.88°, 62.72°, 68.88°, 70.33°, and 75.12°, corresponding to crystallographic planes (101), (004), (200), (105), (211), (204), (116), and (220), respectively while Pt NPs were detected at 2*θ* angles of 47.6° and 54.6° corresponding to (111) and (200), respectively (Attia and Altalhi 2017). The absence of impurity peaks in the XRD pattern indicates that the TiO_2_ nanostructures are pure and well crystalline. Using the Scherer’s equation of the XRD pattern and in agreement with the determination of the average size from TEM images, average crystallite sizes of 7.51 nm for Pt/TiO_2_ NCs and 4.25 nm for NPs were found (Table 1) (Eghbali et al. 2019; Abdelhamid et al. 2020; Attia et al. 2020). Pt/TiO_2_ NCs have a specific surface area of 186.37 m^2^/g, while TiO_2_ NPs have a specific surface area of 225.57 m^2^/g (Table 1).

**Table 1:** Specific surface area, band gap, and XRD data of average size of Pt/TiO_2_ NCs and TiO_2_ NPs

Sample	D_XRD_ (nm)	D_TEM_ (nm)	Lattice constant (Å)		S (m^2^/g)	Band gap (eV)
			a/b	c		
Pt/TiO_2_ NCs	7.51	7.00	3.7754	9.5099	186.37	2.642
TiO_2_ NPs	4.25	4.00	3.7552	9.5098	225.57	3.198

**Fig. 4:**
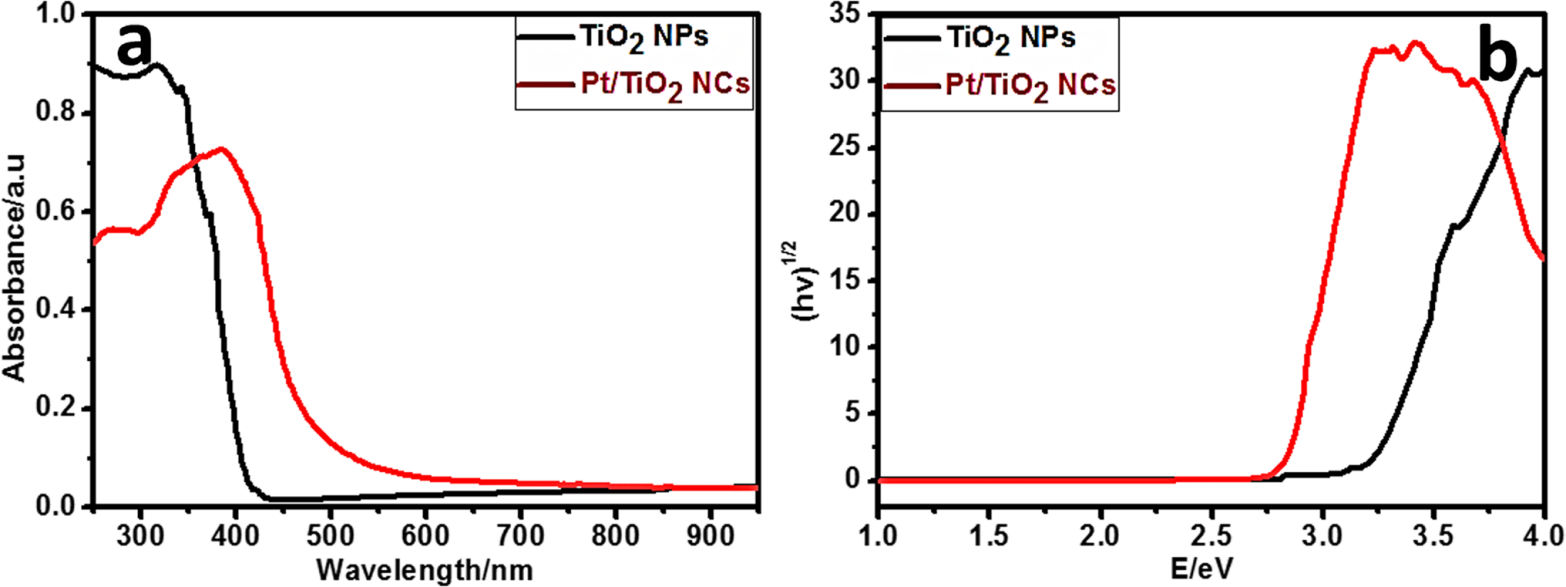
Diffuse reflectance spectra of TiO_2_ NPs and Pt/TiO_2_ NCs

In the region of 200–900 nm, UV–Vis-diffuse reflectance spectra (DRS) were measured (Fig. 4a). The bandgap of the samples was calculated using Regan and Gratzel’s equation (*E*_*g*_ = 1239.8/*λ*), where *E*_*g*_ is the bandgap energy (eV) and (nm) is the wavelength of the absorption edges in the spectra. TiO_2_ NPs have a bandgap of 3.198 eV, while Pt/TiO_2_ NCs have a bandgap of 2.642 eV (Bellardita et al. 2017). Tauc curve of the DRS (Fig. 4b) confirmed the Regan and Gratzel’s calculations.

**Fig. 5:**
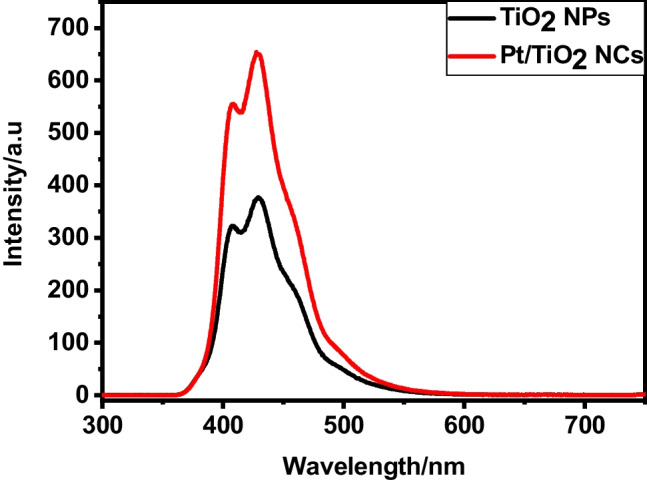
Photoluminescence (PL) emission spectra for TiO_2_ NPs and Pt/TiO_2_ NCs

Photoluminescence (PL) spectra of the TiO_2_ containing Pt-doping in Fig. 5 show that the maximum emission located at 428 nm. TiO_2_ without Pt exhibits about a 45% decrease in luminescence intensity compared with Pt/TiO_2_ NCs. This decrease in PL can confirm the reduction in e–h recombination (Eun et al.^2021^).

**Fig. 6:**
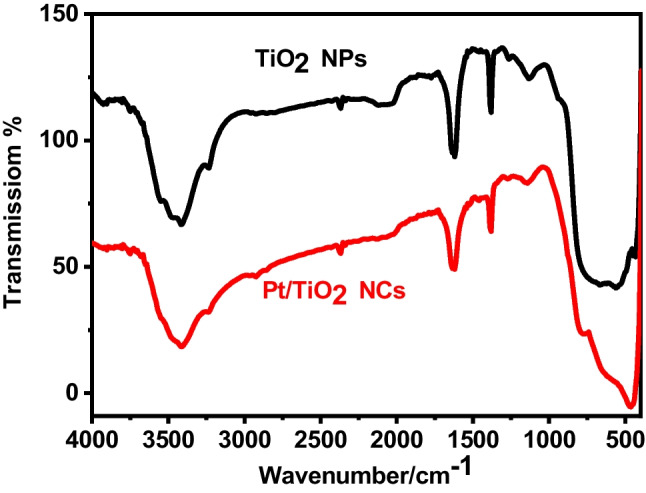
FT-IR spectra of TiO_2_ NPs and Pt/TiO_2_ NCs

The FT-IR spectra of Pt/TiO_2_ NCs and TiO_2_ NPs were shown in Fig. 6. It revealed characteristic peaks at 1628 cm^−1^ and 1386 cm^−1^, which correspond to the vibration of the hydroxyl group of TiO_2_ NPs and the bending mode of water Ti–OH for TiO_2_ NPs. The peaks around 100–1000 cm^−1^ attributed the presence of Pt dopant (Fig. 6) (Wang et al. 2005; Sadeaka et al. 2022).

### Photo-oxidative/transformation of SOx to high valued products

**Scheme 1:**
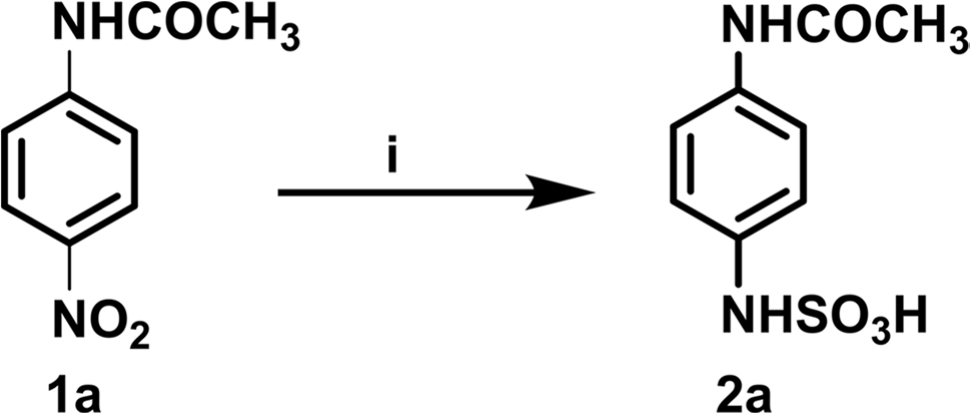
Reaction condition and reagents (i) SOx (1 atm), TiO_2_ NPs or Pt/TiO_2_ NCs, H_2_O: EtOH (1:1, 10 mL), under irradiation of visible light

In our protocol we aimed to investigate the utility of TiO_2_ NPs and Pt/TiO_2_ NCs as detoxification/transformation agents in photocatalytic SOx removal reaction besides the sulfonation of p-nitroacetanilide derivatives **1a** under dark and visible light irradiation conditions. Scheme 1 depicted the reaction between SOx gas at atmospheric pressure with TiO_2_ NPs or Pt/TiO_2_ NCs in the presence of p-nitroacetanilide **1a** under dark and visible light irradiation conditions at room temperature.

By carrying out the reaction under dark conditions, there was no conversion observed, and the organic substance **1a** was recovered without any change. Under the effect of visible light irradiation, the reaction between SOx gas and p-nitroacetanilide **1a** was performed efficiently by using Pt/TiO_2_ NCs rather than the use of TiO_2_ NPs as photocatalyst (Scheme 1). By adding TiO_2_ or Pt/TiO_2_ nanocatalyst and p-nitroacetanilide derivative **1a** (1/3 or 1/2, w/w) to the reaction, followed by interaction of SOx gas (concentration = 10 ppm) through its passing through the reaction solution with 1.0 L/min flow rate, the results showed that Pt/TiO_2_ NCs exhibited higher catalytic activity than TiO_2_ NPs and sulfonated compounds were obtained in high quantitative yields. It was observed that the SOx gas concentration was reduced from 10 ppm to 60 ppb under visible light irradiation which means that the concentration of sulfur dioxide is reduced to 0.6% of the original concentration. The formed sulfamic acid in the solution was analyzed using HR-TOF–MS to measure the % conversion of starting material **1a** product **2a** that was calculated according to the following equation:

$$\%\;\mathrm{Conversion}=\left[\left(C_i-C_f\right)/C_i\right]\times100$$

where *C*_*i*_ is the initial concentration and *C*_*f*_ is obtained concentration after various time intervals.

It was determined that ultrafast conversion of 4-nitroacetanilide was completed for Pt/TiO_2_ within 60 s, while for TiO_2_, it required 200 s, respectively. The highly efficient activity of Pt/TiO_2_ in catalytic applications was attributed to the coexistence of Pt and TiO_2_ nanoparticles in the matrix, which accelerates the electron transfer rate in the catalytic reaction. In addition, smaller bandgap of Pt/TiO_2_ (2.642 eV) (see Fig. 4) was beneficial to improve the charge transfer efficiency in the catalytic process.

As a result, the effectiveness of the removal efficiency of SOx can be elucidated in Table 2 that illustrated the optimized conditions of chemical deSOx according to comparative study in the regard of the catalyst concentration of TiO_2_ NPs or Pt/TiO_2_ NCs as photocatalyst and the reaction time.

**Table 2:** The production yield of products 2a by using TiO_2_ NPs or Pt/TiO_2_ NCs

Product	Catalyst	Concentration catalyst/p-nitroacetanilide (w/w)	Time (s)	% conversion
2a	TiO_2_ NPs	1/3	200	18
		1/2		32
2a	Pt/TiO_2_ NCs	1/3	60	97
		1/2		99

The results showed that Pt/TiO_2_ nanocomposite showed better catalytic performance than TiO_2_ by 3.09 times.

### Recovery and reusability of TiO_2_ and Pt/TiO_2 _nanocatalysts

**Fig. 7:**
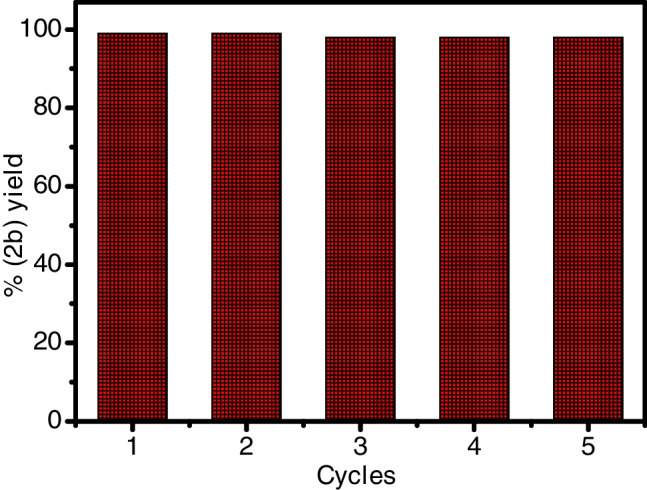
Recovery and reusability of Pt/TiO_2_ nanocatalysts for **2b** production after 10 min under visible light

Pt/TiO_2_ NCs can be easily recovered from the reaction mixture. After completion of the SO_x_ removal reactions by using the aforementioned catalysts, these catalysts were collected from the reaction mixture by flirtation and washed with ethyl acetate, ethanol, and water, and then dried under vacuum. As shown in Fig. 7, Pt/TiO_2_ NCs could be recovered and reused at least five times, without any obvious decrease in catalytic activity.

**Scheme 2:**
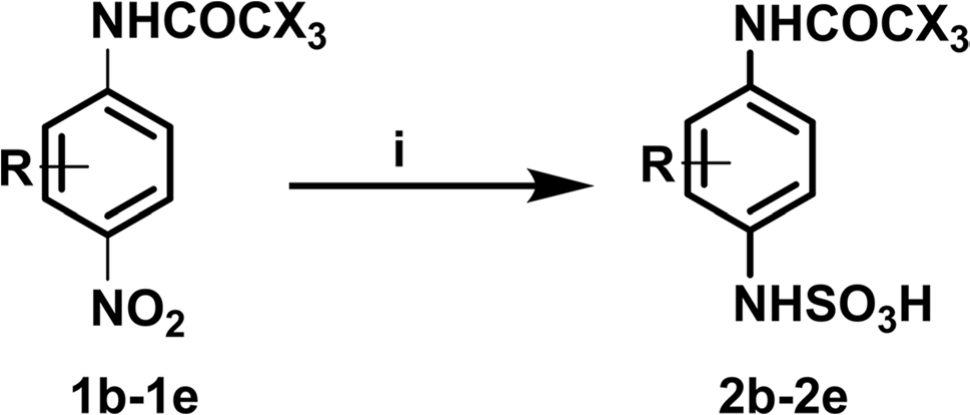
Synthesis of a series of p-aminoacetanilide with the purpose capture of SOx for recovery of Pt/TiO_2_ nanocatalyst from sulfur poising under optimized condition. (i) SOx (1 atm), TiO_2_ NPs or Pt/TiO_2_ NCs (7.5 mg), H_2_O: EtOH (1:1, 10 mL), under irradiation of visible light at 10 min

The limitation and the scope of the method for the removal of SOx as an effective way for recovery of the nanocatalyst from sulfur poising using green and efficient approach at room temperature, under visible light photo-oxidation/photo-reduction approach, were studied. A series of experiments using other p-nitroacetanilide derivatives **2a–2e** was used in the presence of Pt/TiO_2_ NCs under the investigated optimized condition (Scheme 2 and Table 3).

**Table 3:**
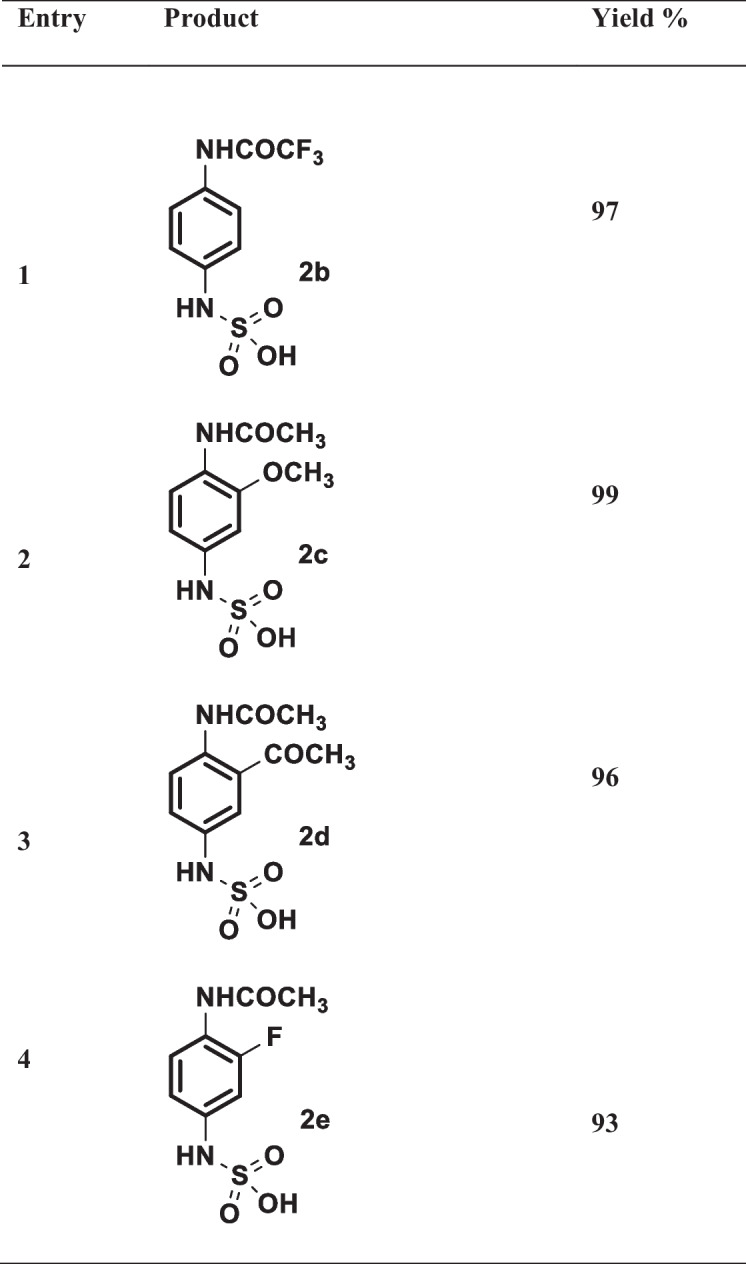
Photocatalytic conversion of 4-acetanilide derivatives to sulfamic acid derivatives

As it was observed from Table 2, several p-nitroacetanilide possessing various functional groups were subjected to the deSOx method. It was investigated that the approach is effective for capturing of SOx by its reaction with p-nitroacetanilide through photo-oxidation/photo-reduction reaction to produce p-aminoacetanilide derivatives **2b–2e**. From the results, it was noted that all the formed products **2a–2e** were afforded in excellent yields 93–99%, respectively without any observable change on the functional groups, i.e., OCH_3_, COCH_3_, F.

## Discussion

**Fig. 8:**
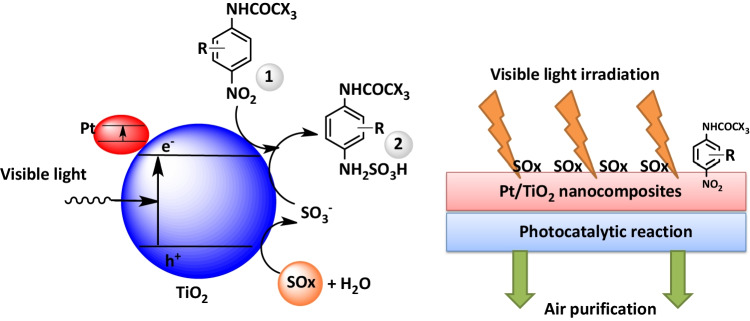
Postulated mechanism for SOx conversion

The photocatalytic removing rate of SOx gas with Pt/TiO_2_ NCs is higher than TiO_2_ NPs under visible light irradiation due to its high specific surface area and narrow bandgap (at visible range). It was indicated that the newly developed protocol has several advantages regarding the formation of series of aromatic sulfonic acids and mitigation of SO_2_ gas effects. From the results, it was demonstrated that Pt/TiO_2_ NCs are known as optical semiconductor which showed greater efficiency than TiO_2_ NPs in absorbing visible light. By subjecting the Pt/TiO_2_ NCs to the energy of visible light, it changes to efficient semiconductor. This happened because electrons in valence band are photoexcited to the conduction band, causing the formation of holes in the valence band (Abdelsalam et al. 2020). The following mechanism can be proposed for the capturing of SOx gases: The noble metal (Pt) absorbs energy from visible light irradiation, as illustrated in Fig. 8.

The electrons of Pt can be excited to its conduction band and then migrate to the conduction band of TiO_2_, followed by electrons can move from the valance band of TiO_2_ to the valance band of Pt due to generated holes in valance band of Pt. The above-mentioned processes can increase the charge carrier lifetime and reduce electron–hole recombination, consequently improving the photocatalytic activity (Alamelu and Ali 2018; Muhich et al. 2012). In the first step, the holes can react with SOx atmosphere producing SO_3_^−^ anions. While in the second step, the excited electrons can react with p-nitroacetanilide derivatives **1** for fixation of the SO_3_^−^ anions forming aromatic sulfonic acid derivatives **2**. These are extremely high beneficial process in which capturing SOx occurred by low-cost aromatic substances and converting them to valued products (Shang et al. 2002).

Table 4 showed comparison between the previous studies and the present approach (Table 4). From these results, it was observed few protocols in literature for abetment SOx through photocatalysis technology using continuous flow reactor. In contrast with other previously reported approaches, the present method was performed under mild condition that assists on efficient deSOx with a facile the synthesis of sulfamic acid derivatives as high-valued pharmaceutical compounds in facile (Şentürk et al. 2012; Abdulrasheed et al. 2018; Berger et al. 2020).

**Table 4:** Previous studies versus the present protocol

Entry	Catalyst	Approach	Concept	Ref.
1	Ti/Al(P1) manifests itself as crystallites of TiO2 on γ-Al_2_O_3_, while Ti/Al(P2) reveals an amorphous AlxTiyOz mixed oxide	Adsorption	The increasing specific surface area upon TiO_2_ promotion showed the superior SOx uptake of Ti/Al(P1, P2) support materials	Şentürk et al. 2012
2	Mesoporous silicas such as SBA-15, MCM-48, MCM-41, MSU-H, and KIT-6	Adsorption	These catalysts acquired large surface area and large pore volume	Abdulrasheed et al. 2018
3	CuO/SBA-15 at	Regenerable solid sorbent materials	The chemisorption of SO_3_ as CuSO_4_, which can be converted back to CuO under a relatively mild thermal treatment (400 °C)	Berger et al. 2020
4	Pt/TiO_2_	Photocatalysis with adsorptive process	The conversion of SO_2_ to SO_3_ oxidation and SO_2_/SO_3_ trapping through production of sulfamic acid	In the present study

## Conclusions

In this study, Pt/TiO_2_ NCs and TiO_2_ NPs photocatalysts were prepared and used for ultrafast reduction of 4-nitroacetanilides using flow photocatalytic reactor. The reaction of SOx as sulfonating agent with 4-nitroacetanilide derivatives under visible light irradiation conditions for the formation of sulfamic acid derivatives was investigated. Full characterizations of the as-prepared nanocatalysts were also conducted to investigate the relationship between the physico-chemical properties and the photocatalytic activity. The study showed that online continuous flow reactor–HRMS-TOF system was constructed to realize real-time monitoring of the reaction time. In this system, nearly complete reaction of 4-nitroacetanilide was achieved in a short time (60 s). This approach of SO_x_ photocatalytic conversion is characterized by high performance, no need for high temperature or pressure, and the catalytic removal carried out in a short reaction time.

## Supplementary Information

Below is the link to the electronic supplementary material.

Supplementary file1 (DOCX 937 KB)

## Data Availability

The dataset supporting the conclusions of this article is available in the [https://pubs.rsc.org/en/content/articlelanding/2017/sc/c6sc03500k] repository.
