# Supplementary material for: Nano Pt/TiO2 photocatalyst for ultrafast production of sulfamic acid derivatives using 4-nitroacetanilides as nitrogen precursor in continuous flow reactors
Source: Environ Sci Pollut Res Int. 2023 Feb 21;30(17):51344–55. doi: 10.1007/s11356-023-25968-9 (PMC10104933; doi:10.1007/s11356-023-25968-9)
Supplement: Supplementary file 1 — Supplementary file1 (DOCX 937 KB) [file 11356_2023_25968_MOESM1_ESM.docx]

**Supporting information**

**Nano Pt/TiO_2_ Photocatalyst for Ultrafast Production of Sulfamic Acid Derivatives Using 4-Nitroacetanilides as Nitrogen Precursor in Continuous Flow Reactors**


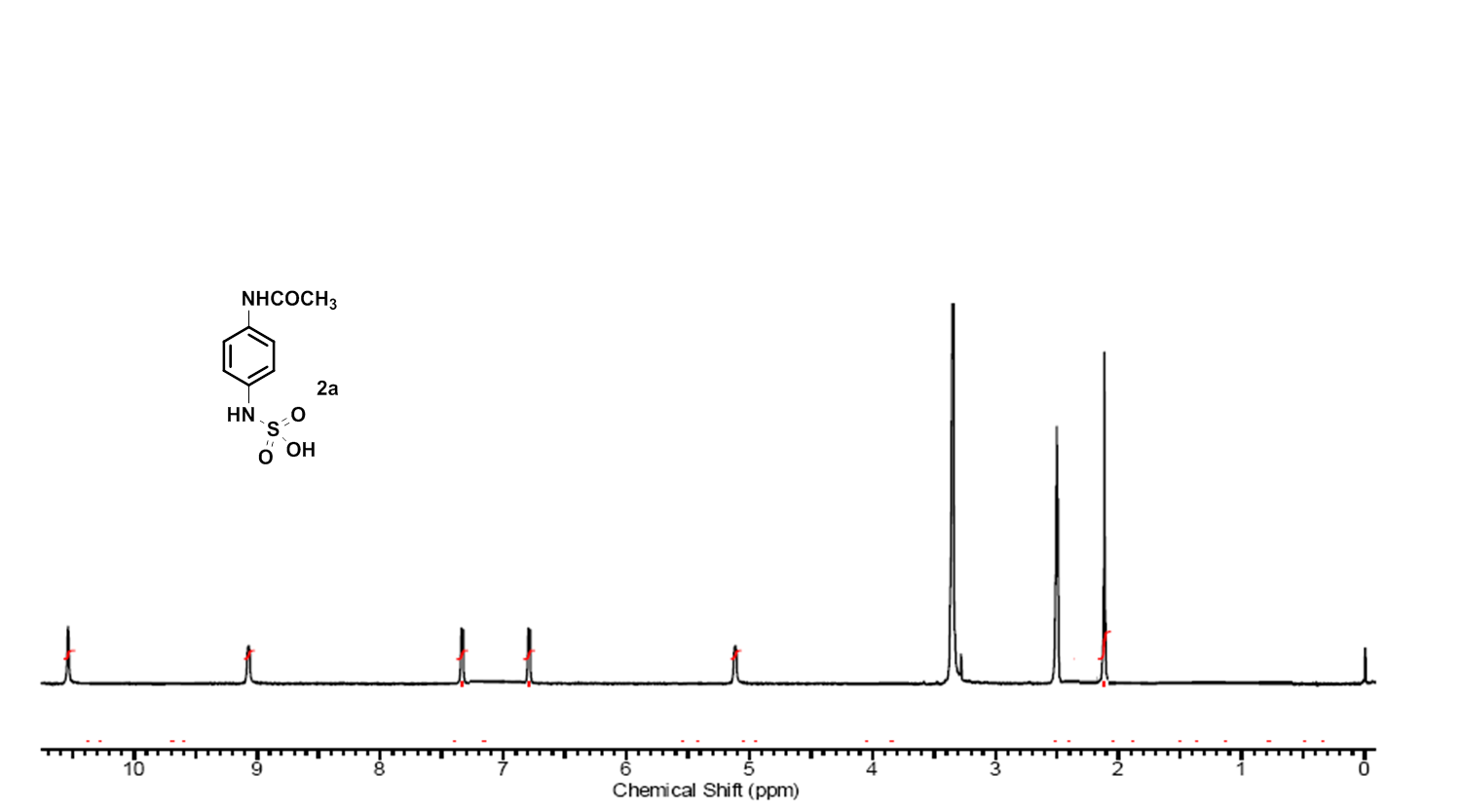


^1^H NMR of compound **2a**


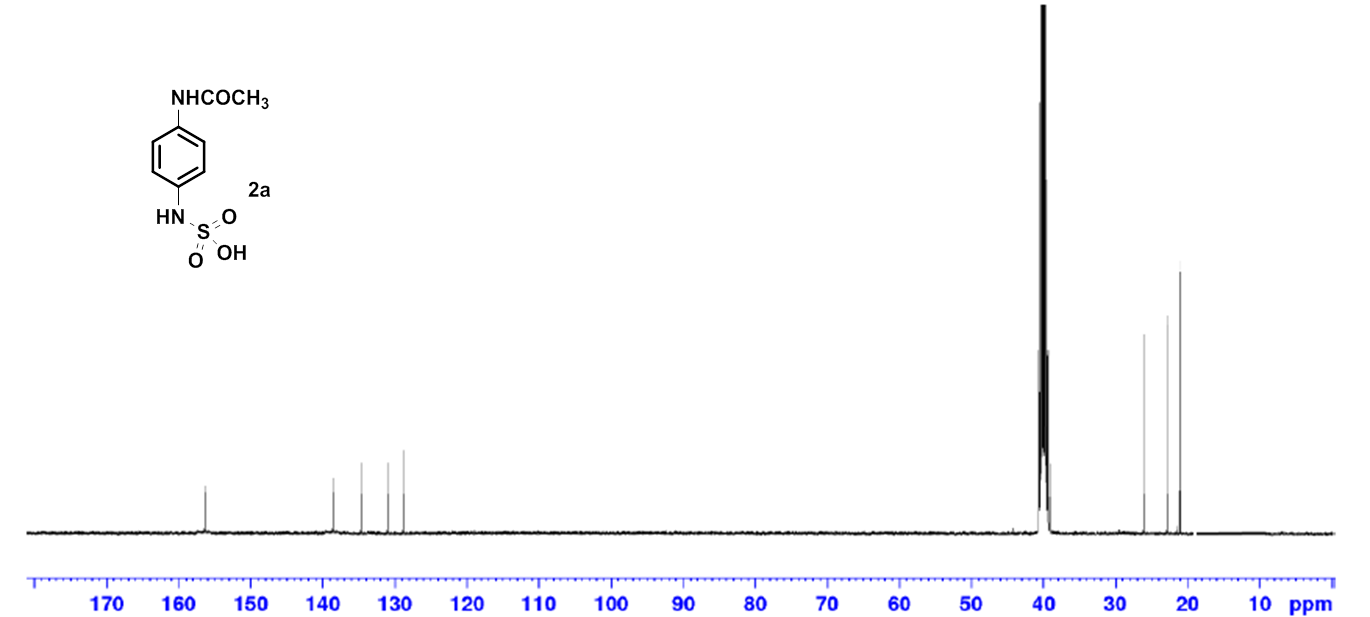


^13^C NMR of compound **2a**


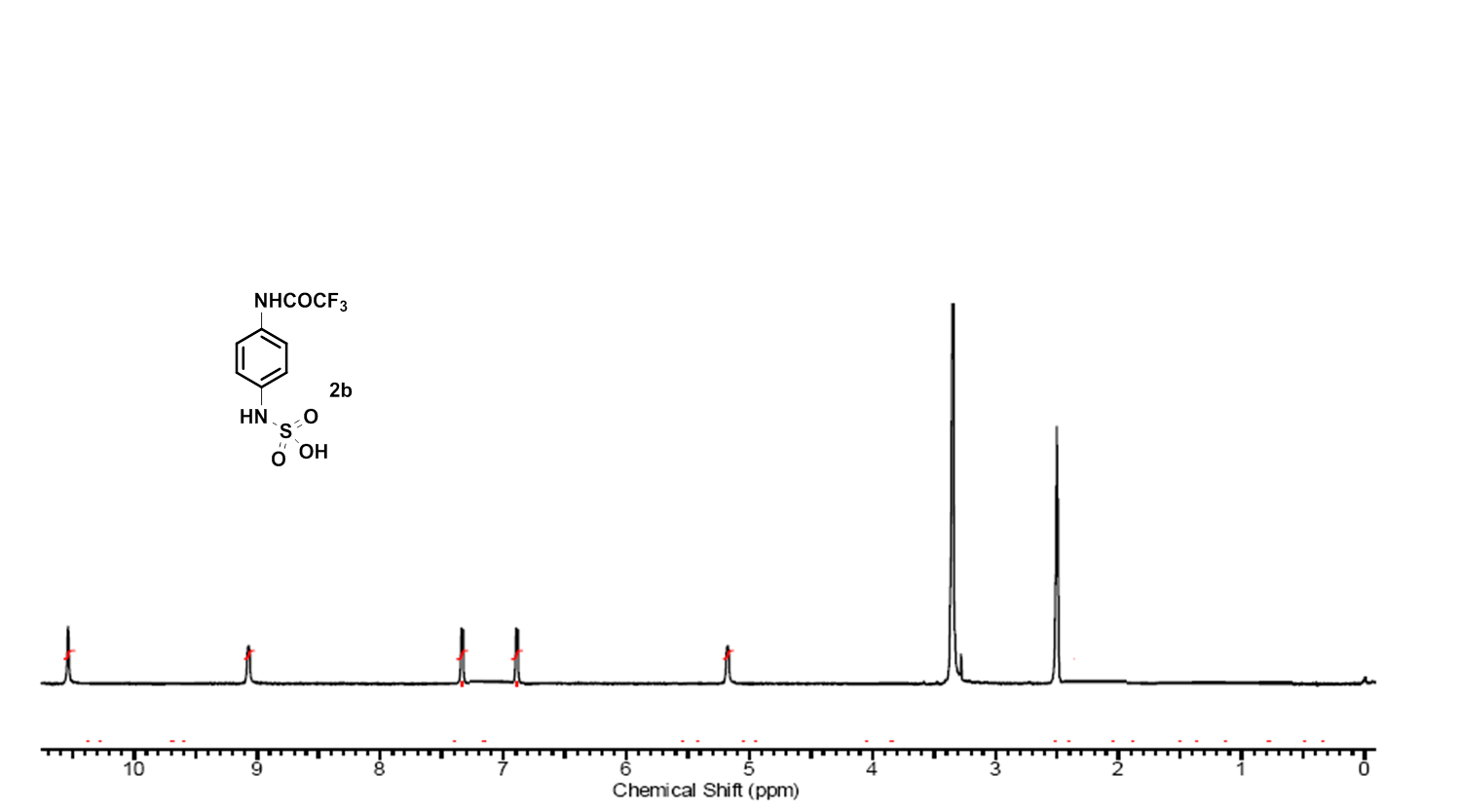


^1^H NMR of compound **2b**


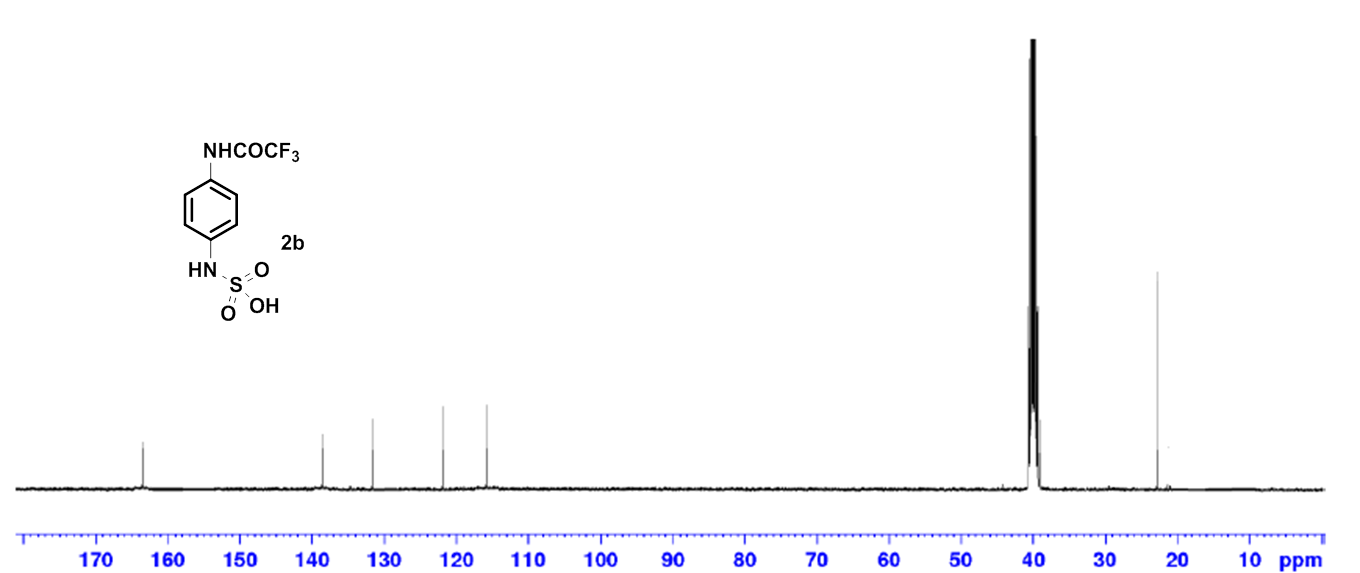


^13^C NMR of compound **2b**


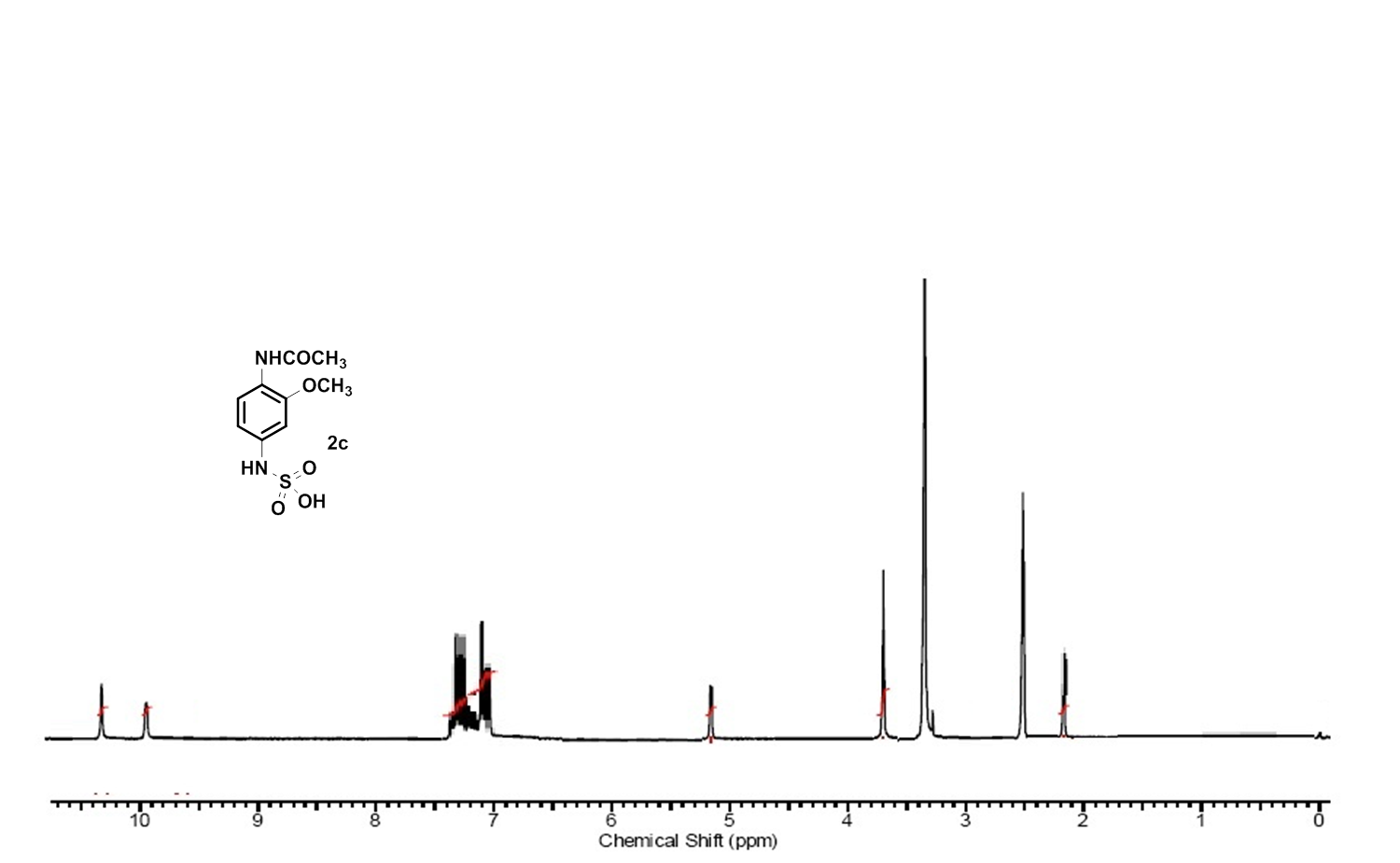


^1^H NMR of compound **2c**


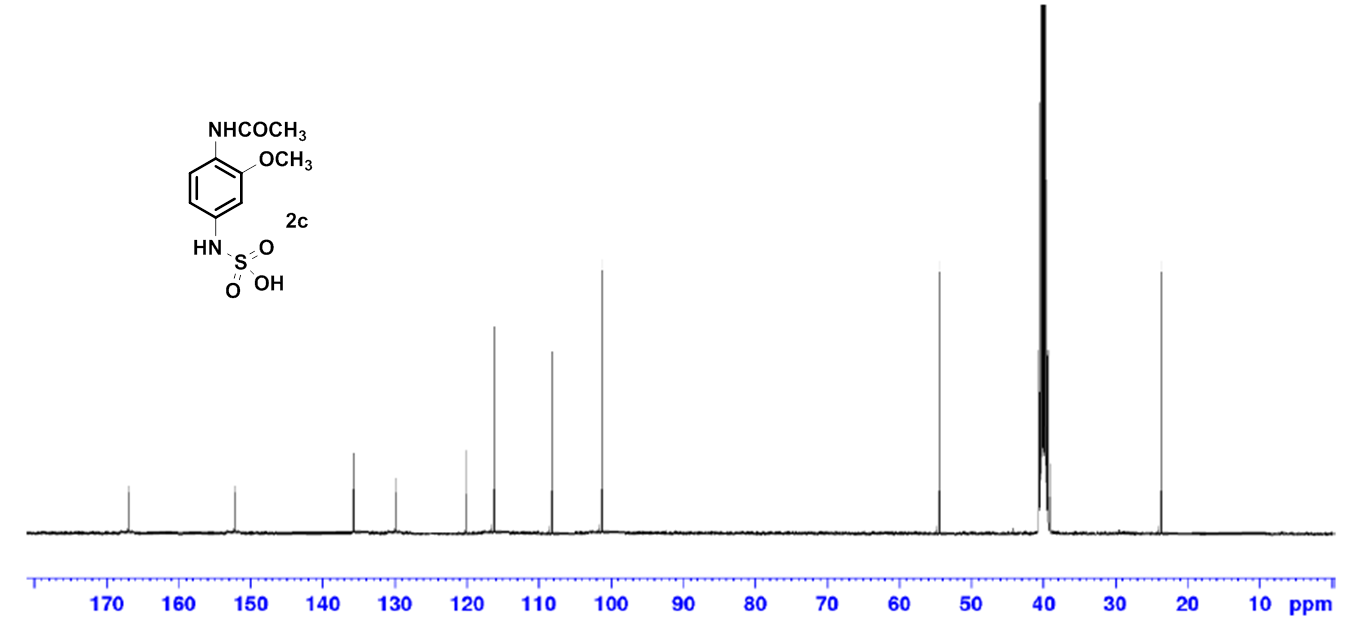


^13^C NMR of compound **2c**


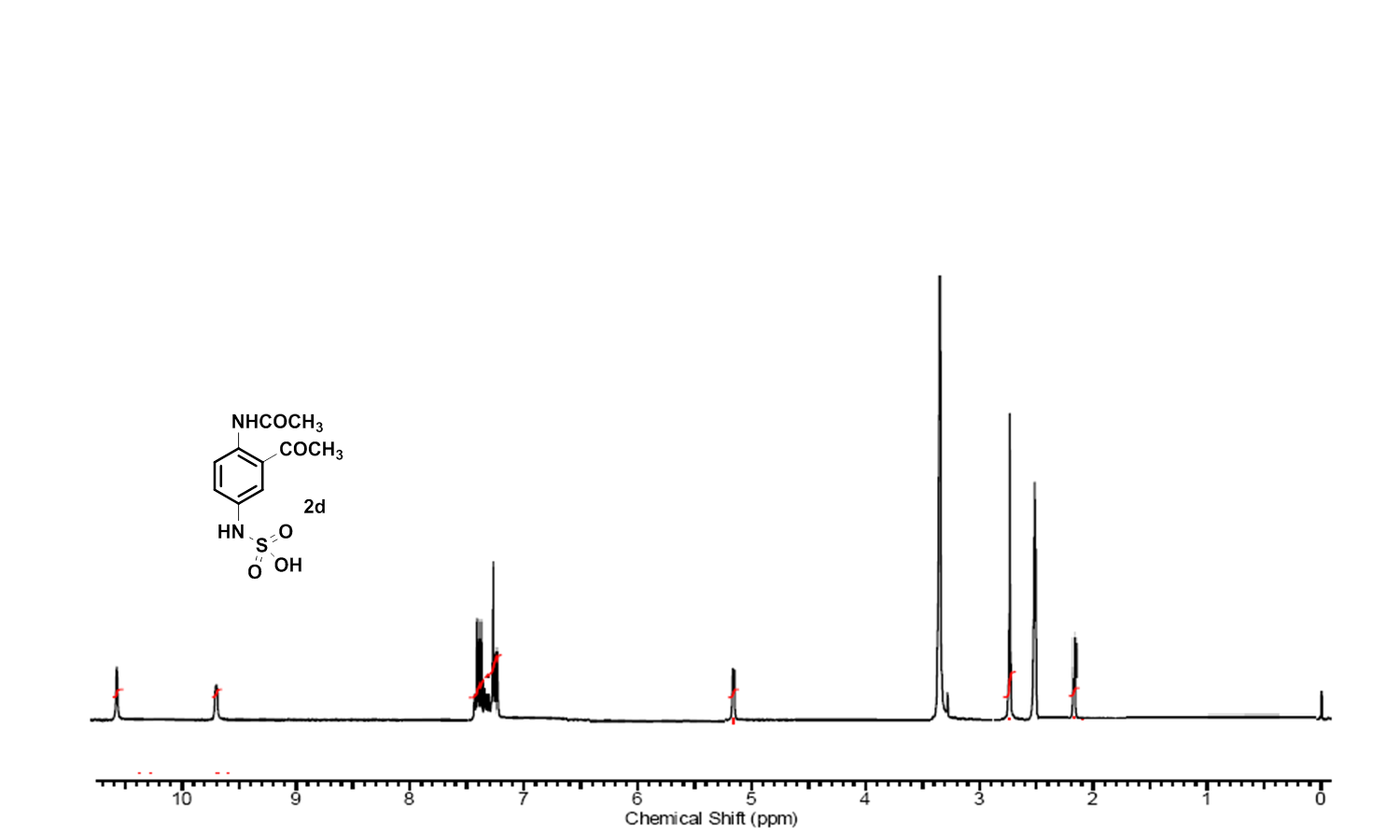


^1^H NMR of compound **2d**


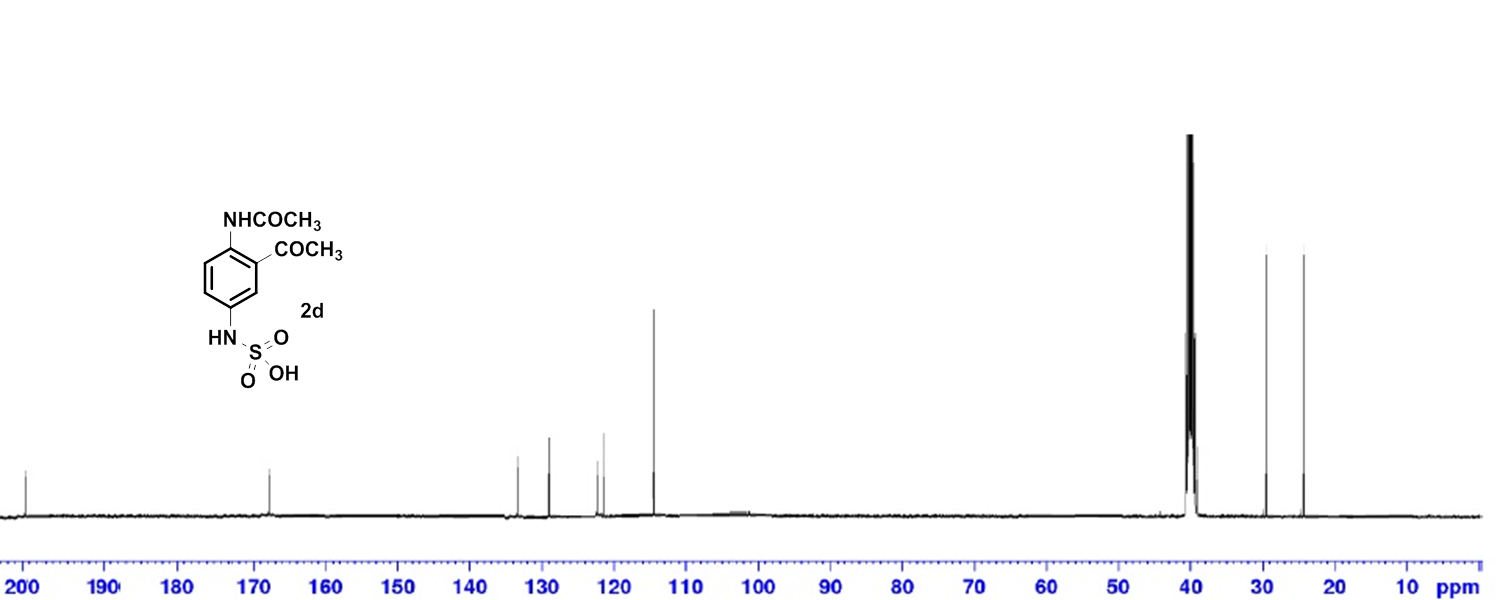


^13^C NMR of compound **2d**


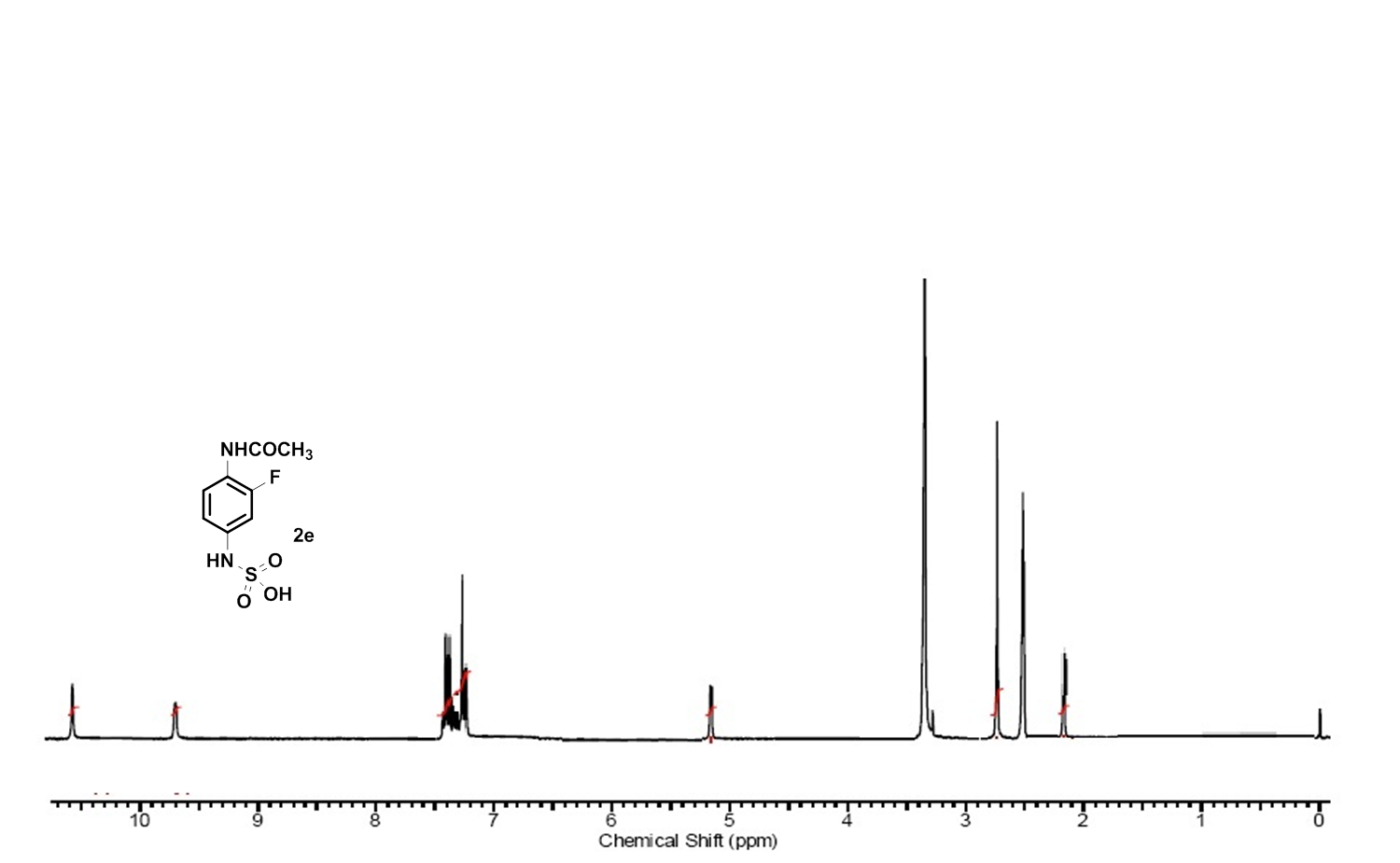


^1^H NMR of compound **2e**


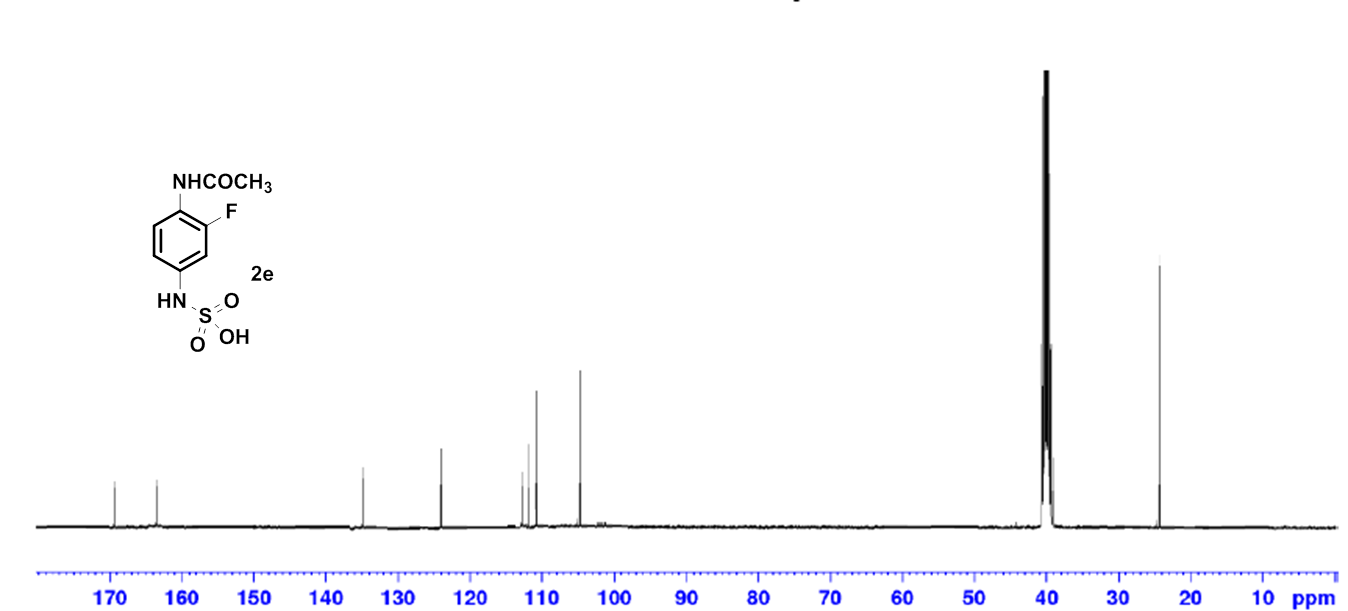


^13^C NMR of compound **2e**
